# Effects of an antiatherogenic diet during pregnancy on markers of maternal and fetal endothelial activation and inflammation: the CARRDIP study

**DOI:** 10.1111/j.1471-0528.2006.01187.x

**Published:** 2007-01-08

**Authors:** J Khoury, T Henriksen, I Seljeflot, L Mørkrid, KF Frøslie, S Tonstad

**Affiliations:** a Department of Obstetrics and Gynaecology, Rikshospitalet-Radiumhospitalet Medical Centre Oslo, Norway; b Department of Cardiology, Centre for Clinical Research, Ullevaal University Hospital Oslo, Norway; c Department of Clinical Chemistry, Rikshospitalet-Radiumhospitalet Medical Centre Oslo, Norway; d Section of Biostatistics, Rikshospitalet-Radiumhospitalet Medical Centre Oslo, Norway; e Department of Preventive Cardiology, Ullevaal University Hospital Oslo, Norway

**Keywords:** Diet, inflammation, pregnancy, preterm delivery

## Abstract

**Objective:**

To study the effect of an antiatherogenic diet on maternal and cord blood concentrations of systemic biomarkers of endothelial cell activation, haemostasis and inflammation.

**Design:**

Single blinded randomised controlled clinical trial.

**Setting:**

Obstetric outpatient clinic and maternity unit of a university hospital in Norway.

**Population:**

Nonsmoking pregnant women aged 21–38 years carrying a single fetus and with no previous pregnancy-related complications.

**Methods:**

Subjects (*n* = 290) were randomised to continue their usual diet or to adopt a diet low in saturated fat and cholesterol from gestational week 17–20 to birth. Soluble forms of cellular adhesion molecules, high-sensitivity C-reactive protein (CRP) and haemostatic markers were measured at 17–20 weeks of gestation (baseline) and subsequently up to week 36. All the above, except CRP, were also measured in cord blood.

**Main outcome measures:**

Concentrations of maternal and fetal biomarkers and maternal CRP.

**Results:**

All biomarkers except CRP levels increased significantly during the study period in both the intervention and control groups. None of the maternal or fetal biomarkers were influenced by the intervention (*P* > 0.05) except for a tendency to lower concentrations of cord blood tissue plasminogen activator antigen in the intervention group compared with the control group, median (interquartile range) 5.4 ng/ml (3.1–7.7) versus 5.8 ng/ml (3.5–11.8), *P* = 0.05.

**Conclusion:**

An antiatherogenic diet in pregnancy did not significantly influence maternal or fetal blood concentrations of a range of biomarkers for inflammation. Thus, the previously reported effects of a cholesterol-lowering diet on maternal lipid profile and preterm delivery (<37 complete weeks of gestation) do not seem to involve changes in the systemic inflammatory responses of pregnancy.

## Introduction

Complications of pregnancy, including pre-eclampsia, spontaneous loss of early pregnancy, low birthweight and preterm delivery, have been linked to the maternal risk of coronary heart disease in later life.^1^,^2^ Pathological changes in the uteroplacental circulation in pre-eclampsia and preterm delivery share common features with atherosclerosis.^3^,^4^ Adverse pregnancy outcomes are also related to the infant’s cardiovascular risk in later life.^5^,^6^ Moreover, maternal hypercholesterolaemia is associated with fetal and childhood atherosclerosis.^7^,^8^

The Cardiovascular Risk Reduction Diet in Pregnancy (CARRDIP) trial was a randomised clinical trial designed to assess the effect of a low-cholesterol, low-saturated-fat antiatherogenic diet compared with the usual diet among nonsmoking white European healthy women on maternal, cord and neonatal lipid concentrations, and on pregnancy outcome. Maternal lipid concentrations were reduced in the intervention arm of the trial, while cord blood and neonatal lipids did not differ between the groups. The diet had no measured adverse effects. However, there was a marked reduction in the incidence of preterm delivery (a live birth prior to 37 complete weeks of gestation) in the intervention group. Gestational age was based on the ultrasound examination performed at week 17–18 of gestation. There were no differences between the intervention and control groups with respect to other pregnancy complications.^9^

Inflammatory mechanisms are involved in atherogenic processes as well as in several pregnancy complications including preterm delivery.^10^–^13^ These mechanisms may be reflected in elevated blood concentrations of soluble adhesion molecules (sCAMs), markers of inflammation and endothelial activation, such as vascular adhesion molecule (VCAM-1) and intercellular adhesion molecule (ICAM-1), as has often been observed in pre-eclampsia.^14^–^23^ On the other hand, the concentration of high-sensitivity C-reactive protein (CRP), though emerging as a potential marker for cardiovascular risk,^24^ has not been shown to differ significantly in women who subsequently develop pre-eclampsia or fetal growth restriction compared with women with uncomplicated pregnancies.^18^,^19^ Furthermore, markers of prothrombotic activity like von Willebrand’s factor (vWF) and markers of abnormal fibrinolysis including plasminogen activator inhibitor type 1 (PAI-1 activity), PAI type 2 (PAI-2 antigen) and tissue plasminogen activator antigen (tPAag) have been identified as independent markers of cardiovascular risk in several studies^25^–^27^ and have also been related to pregnancy outcome.^20^–^23^

Several studies in nonpregnant subjects have indicated that diet influences concentrations of sCAMs and other novel cardiovascular risk markers 28–30 but have not been studied in relation to dietary intervention in pregnancy. In this analysis of the CARRDIP population, we investigated the effect of an antiatherogenic diet on maternal and fetal (cord blood) concentrations of sCAMs, markers of thrombosis and fibrinolysis and on maternal levels of high-sensitivity CRP.

## Methods

The trial has been described in detail previously.^9^ In brief, 290 healthy, nonsmoking, pregnant white European women aged 21–38 years, without a previous or current pregnancy complication and carrying a single healthy fetus, were randomised to follow their usual diet or the intervention diet. The randomisation list was generated from a table of random numbers drawn up by the investigator (S.T.) who had no contact with the pregnant women (as described previously).^9^ Sealed opaque envelopes consecutively numbered and containing the randomisation number and code (control or intervention) were given to the dietician responsible for giving the dietary advice. Thereafter, each screened and eligible woman was allocated the next available number by the dietician who opened the envelope to determine whether the woman was to follow the usual diet or the intervention diet.

**Figure 1:**
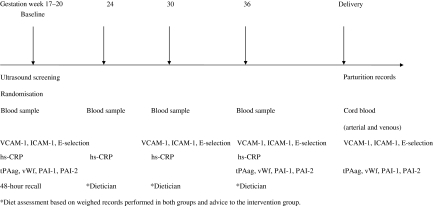
Assessment and scheduling procedures.

The intervention diet aimed to limit dietary cholesterol and to reduce the intake of saturated fat by replacing saturated fat with monounsaturated and polyunsaturated fat. Women in the intervention group (*n* = 141) were advised to consume fish, low-fat meats, oils and low-fat dairy products instead of full-fat dairy products and meats, and to increase their intake of whole grains, fruits, vegetables and legumes. Both the intervention and control groups followed the same schedule of blood sampling, physician and dietician visits and assessments (Figure 1). Usual antenatal care was provided by the woman’s general physician and/or midwife. The initial and all the follow-up examinations pertaining to the study were conducted by a specialist in obstetrics and gyanecology (J.K.). The physicians and midwives caring for the women, the specialist physician (J.K.) and all laboratory and other personnel were blinded with regard to treatment assignment. The subjects were asked not to reveal their dietary assignment to any of the study staff or other subjects.

To assess dietary compliance, the weighed dietary intake was recorded on a predetermined day weekly throughout pregnancy. The women were instructed in the cumulative weighing technique using a digital dietary scale. All food and beverages consumed were described and weighed. The dietary records were entered into the database by certified personnel and scored using the software program Beregn developed by the Department of Nutrition, University of Oslo, and based on the Norwegian food table (1995; National Nutrition Council). Foods or recipes that were not part of the programme were calculated and added. Vitamin or iron supplements were not included in the analyses.

### Laboratory methods

Blood samples for measuring the risk markers (hereinafter referred to as biomarkers) were obtained at each visit after an overnight fast of at least 10 hours. VCAM-1, ICAM-1 and E-selectin were measured at 17–20 weeks of gestation (baseline) and at weeks 30 and 36. High-sensitivity CRP was measured at baseline and at weeks 24, 30 and 36. PAI-1, PAI-2, tPAag and vWF were measured at baseline and week 36. All the above, except CRP, were also measured in cord blood. Serum separated within 1 hour was used for determination of VCAM-1, ICAM-1, E-selectin and CRP. Blood for the preparation of platelet-poor citrated plasma was collected, immediately placed on ice and centrifuged within 30 minutes (maternal samples) to 1 hour (cord blood samples) for determination of PAI-1, PAI-2, tPAag and vWF. All samples were kept frozen at −70°C until analysed.

Commercial methods were used for all analyses. sCAMs were analysed with kits provided by R&D Systems Europe, Abingdon, Oxon, UK, and the individual CAMs had the following coefficients of inter-assay variation (CV): VCAM-1, 5.2%; ICAM-1, 4.8%; and E-selectin, 5.3%. High-sensitivity CRP was measured by means of the Roche Modular electrochemoluminescence method. CVs were 8.0, 4.0 and 6.0% at concentrations of 0.13, 1.18 and 17.0 mg/l, respectively. Commercial methods were also used for analyses of the haemostatic parameters as follows: Spectrolyse/pL PAI-1 (Biopool AB, Umeå, Sweden; CV 4.2%), Imubind PAI-2 (American Diagnostica Inc., Greenwich, CT, USA; CV 6.3%), TintElize tPAag (Biopool AB; CV 3.5%) and Asserachrom vWF (Stago Diagnostica, Asnieres, France; CV 8.0%).

Fatty acid measurements were carried out in serum frozen immediately after venesection and stored at −70°C. A single technician analysed all samples simultaneously after completion of the trial. Serum total fatty acid profiles were determined at baseline and at gestational week 36 by extracting lipids from serum. After transesterification, fatty acid methyl esters were separated by means of capillary gas liquid chromatography. The identity of each fatty acid peak was ascertained by comparison of the peak’s retention time relative to the retention times of fatty acids in a synthetic standard of known fatty acid composition. The relative amount of each fatty acid (% of total fatty acid) was quantified by integration of the area under the peak and dividing the result by the total area for all fatty acids. All the analyses were performed at the Department of Clinical Chemistry, National Hospital, Oslo, under the supervision of Bjørn Christophersen.

### Statistical analyses

The assessment of the effects of an antiatherogenic diet on markers of maternal and fetal endothelial activation and inflammation was part of the secondary hypotheses of the CARRDIP study and part of the original protocol. The power calculations were, however, based on our primary hypothesis concerning the expected difference in maternal total cholesterol levels at week 36 and neonatal total cholesterol levels in the intervention versus the control groups.^9^ In brief, a sample size of 130 women in each group was required to detect a 5% reduction in maternal total cholesterol and a change of 15% (about 0.22 mmol/l) in neonatal total cholesterol levels between the two groups, (*α* = 0.05; *β* = 0.20) in the intervention versus the control groups. A total inclusion of 300 participants was planned in case of dropouts. With an expected rate of adverse pregnancy outcome of 25% (data based on the Norwegian Birth Register), this number of participants had about 65% power with *α*set at 0.05 to find a 50% increase in the occurrence of any adverse pregnancy outcome.

Dietary energy and intake of macronutrients and food stuffs during pregnancy were expressed as mean daily intake, based on weighed recordings of intake from baseline to week 36.

All group differences (intervention versus control group), including baseline characteristics, were analysed by two-sample *t*tests or Mann–Whitney *U*test, as appropriate. The tests used are shown in the tables. The tPAag concentrations in cord blood did not follow a normal distribution; hence, a Mann–Whitney *U*test was used. However, due to the numerical differences shown in the percentiles (Table 4), an additional analysis based on the lognormal transformation of tPAag was carried out. The transformed data were compared by a two-sample *t*test.

**Table 4:** Concentrations of markers of endothelial activation and prothrombotic activity in cord blood in the control and intervention groups

	No. control/intervention	Control	Intervention	*P*value*
VCAM-1 (ng/ml)	120/119	813 (695, 970)	836 (715, 993)	0.4
ICAM-1 (ng/ml)	120/119	149 (130, 167)	148 (128, 174)	0.7
E-selectin (ng/ml)	120/118	150 (96.7, 189)	159 (123, 201)	0.08
tPAag (ng/ml)	119/117	5.8 (3.5, 11.8)	5.4 (3.1, 7.7)	0.2
vWF (%)	107/104	91 (66, 135)	82 (54, 120)	0.3
PAI-1 activity (u/ml)	118/115	9.3 (4.6, 17.8)	10.3 (5, 19)	0.6
PAI-2 antigen (ng/ml)	119/114	3.4 (1.1, 9.4)	3.7 (1.9, 6.7)	0.6

Values shown are median (25, 75 percentiles).

* Mann–Whitney *U*test for group differences.

Boxplots were used to describe the concentrations of the biomarkers at different time points during the study period. CRP values higher than 10 mg/l were excluded because of a number of confounding causes of elevated CRP.^24^ The number of excluded cases due to elevated CRP in the control and intervention groups, respectively, were as follows: *n* = 24 and 11 at baseline, *n* = 10 and 12 at week 24, *n* = 15 and 10 at week 30 and *n* = 11 and 11 at week 36. The Wilcoxon test for paired samples was used to test changes from baseline to week 36 within the intervention and control groups and in the cohort as a whole.

Pearson’s correlation coefficient was used to assess correlation between baseline CRP and baseline body mass index (BMI) in the entire cohort before randomisation. All analyses were performed using the software program SPSS version 12.0. A two-sided *P*value <0.05 was considered statistically significant.

## Results

The intention-to-treat cohort included 290 women. Of these, 149 were randomised to continue their usual diet (control) and 141 were randomised to the intervention diet. Main baseline characteristics of the participants are shown in Table 1. No differences in baseline characteristics or dietary variables between the groups were observed before randomisation.^9^

**Table 1:** Characteristics of participants at baseline

Characteristic	Control	Intervention	*P*-value
No.	149	141	
Age (years)	29.8 (3.4)	29.6 (3.7)	0.7
Nulliparous (%)	65.1	70.9	0.3
Gestational age at entry (week)	19 (1.1)	19 (1.1)	0.9
	19 (5.4)*	19 (4.9)*	
BMI (kg/m^2^)	24.3 (2.7)	24.3 (2.9)	0.9
BMI (%) >25 (kg/m^2^)	32.9	36.2	0.6
Systolic blood pressure (mmHg)	105 (10)	106 (9)	0.9
Diastolic blood pressure (mmHg)	65 (8)	64 (8)	0.4

Values are mean (SD).

* Median (range).

### Dietary adherence

Based on dietary records of weighed intake from baseline to gestation week 36, the intervention group consumed less energy and total fat than the control group.^9^ This was due to a lower intake of saturated fat and dietary cholesterol.^9^ Compared with the control group, the intervention group significantly reduced their intake of fatty milk and meat products and significantly increased their intake of fish and fish products. Mean dietary intake of fatty fish and fish products during the study period was also significantly higher in the intervention group compared with the control group (Table 2). In addition, the intervention group increased their use of monounsaturated oils, mainly olive oil and rapeseed oil. Moreover, the consumption of nuts, olives, seeds, fruits and vegetables was significantly higher in the intervention group compared with the control group (Table 2).

**Table 2:** Mean (SD) daily dietary intake from weighed food records during the study period

	Control diet (*n* = 141)	Intervention diet (*n* = 127)	*P*value
**Total energy (kcal/day)**	2189 (335)	2031 (348)	<0.001
**Total fat (% of energy)**	32.3 (3.4)	30.0 (4.1)	<0.001
**Protein (% of energy)**	14.7 (1.5)	15.6 (1.6)	<0.001
**Carbohydrates (% of energy)**	52.3 (3.7)	53.7 (4.3)	0.004
Sugar (% energy)	11.6 (4)	9.1 (4.3)	<0.001
**Milk and milk products (g/day)**	457 (172)	415 (165)	0.05
Fatty milk (g/day)	27 (40)	14.2 (18.1)	<0.001
Cheese (g/day)	37.1 (16.2)	33.2 (17)	0.07
**Meat and meat products (g/day)**	91.5 (28.9)	75.2 (27.8)	<0.001
**Fatty minced meat (g/day)**	7.5 (9)	3.6 (6.1)	<0.001
**Fish and fish products (g/day)**	48.6 (27.1)	61.8 (24.5)	<0.001
Fatty (fish and products)	16.0 (14.6)	25.2 (16)	<0.001
**Butter (g/day)**	3.8 (4.7)	0.97 (2.1)	<0.001
**Hard margarines (g/day)**	1.0 (1.4)	0.4 (1.0)	<0.001
**Soft margarines (g/day)**	16.6 (8.1)	16.1 (9.4)	0.7
Rapeseed-based margarine	0.97 (2.3)	6.2 (5.1)	<0.001
**Oils (g/day)**	3.6 (5.1)	8 (4.8)	<0.001
Olive oil	1.7 (2.5)	6.8 (4.5)	<0.001
Rapeseed oil	0.3 (1.0)	1.4 (1.8)	<0.001
**Nuts, olives and seeds (g/day)**	3.7 (5.1)	7.6 (7.5)	<0.001
**Vegetables (g/day)**	139 (57.5)	165 (66.5)	0.001
**Fruits (g/day)**	407 (147)	471 (171)	0.002

T-test for group differences.

The increase in myristic acid (C14:0) level in serum total fatty acids from baseline to week 36 was significantly lower in the intervention group compared with the control group, while eicosapentaenic acid (C20:5) levels tended to increase more in the intervention than in the control group (Table 3).

**Table 3:** Fatty acid composition (% of total serum fatty acids) at baseline and week 36

	Control (*n* = 129)		Intervention (*n* = 125)		P*
			
	Baseline	Week 36	Baseline	Week 36	
C14:0	0 (0, 0)	0 (0, 0.7)	0 (0, 0)	0 (0, 0.6)	0.02
C14:0**	0.15 (0.33)	0.35 (0.39)	0.18 (0.33)	0.28 (0.38)	
C16:0	26.5 (25.3, 28.1)	29.5 (28.1, 31.4)	26.5 (25.2, 27.9)	29.3 (27.5, 31.0)	0.4
C18:0	7.5 (6.7, 8.1)	7.2 (6.3, 7.8)	7.6 (6.9, 8.1)	7.0 (6.5, 7.6)	0.1
C18:1	21.1 (19.6, 22.5)	21.8 (20.3, 24.0)	20.7 (19.2, 22.2)	22.2 (20.5, 24.2)	0.3
C18:2	27.6 (25.4, 29.9)	25.6 (24.3, 28.6)	27.7 (25.3, 30.6)	26.3 (24.4, 28.6)	0.7
C18:3	0.7 (0, 0.8)	0.7 (0.5, 0.8)	0.7 (0, 0.8)	0.7 (0, 0.8)	0.8
C20:4	7.0 (6.1, 7.8)	5.9 (5.2, 6.7)	6.9 (6.2, 7.9)	5.9 (5.1, 6.7)	0.1
C20:5	0.9 (0.4, 1.4)	0.6 (0, 1.0)	1.0 (0, 1.5)	0.7 (0,1.4)	0.06
C22:6	7.6 (5.9, 9.6)	6.5 (4.7, 8.5)	7.6 (5.9, 9.4)	6.7 (5.0, 8.5)	0.7

Data are shown as median (25,75 percentiles), except for C14:0 where

** mean (SD) values are supplemented.

* Mann–Whitney *U*test for the change from baseline to week 36.

### Maternal biomarkers and the dietary intervention

**Figure 2:**
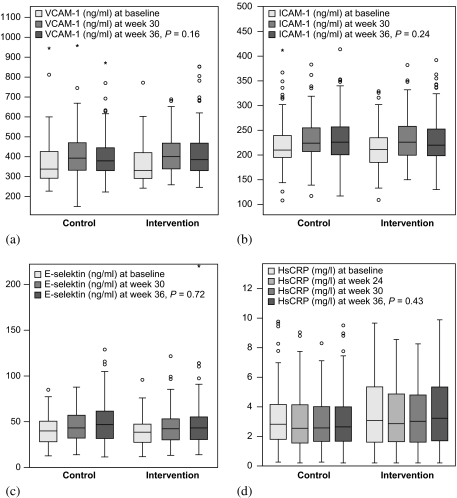
Boxplot for the concentrations of sCAMs and high-sensitivity (Hs) CRP (CRP values >10 not included) during the study period in the control and intervention groups. *P*values refer to group differences expressed as changes in those markers from gestation week 17–20 (baseline) to gestation week 36.

**Figure 3:**
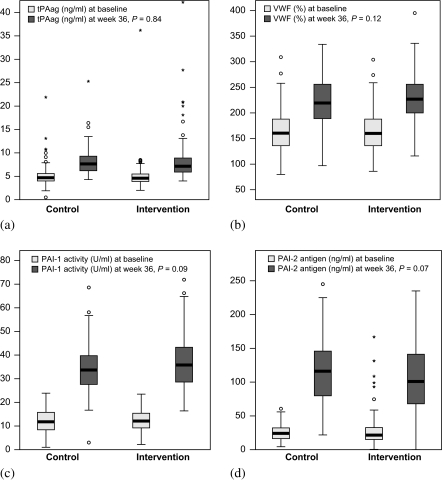
Boxplot for the concentrations of the haemostatic markers (markers of prothrombotic activity) during the study period in the control and intervention groups. *P*values refer to group differences expressed as changes in the haemostatic markers from gestation week 17–20 (baseline) to gestation week 36.

Over the duration of the study period (between baseline and week 36), there was a significant increase in the concentrations of sCAMs and the haemostatic markers both within the intervention and control groups analysed separately (Figures 2 and 3; *P* < 0.01 for all variables) and within the entire cohort (data not shown; *P* < 0.01 for all variables). However, the concentrations of CRP did not increase within the intervention and control groups analysed separately (Figure 2d, *P* > 0.1) or as an entire cohort (data not shown, *P* > 0.1). Concentrations of sCAMs, CRP and the haemostatic markers did not differ between the control and intervention groups (Figures 2 and 3, *P*values are shown with each boxplot and refer to group differences for changes in those markers from gestation week 17–20 [baseline] to gestation week 36). In the total study population (control and intervention groups combined), a positive significant correlation was found between baseline CRP and baseline BMI (*r* = 0.27, *P* = 0.01).

### Biomarkers in cord blood

Concentrations of fetal sCAMs and haemostatic markers in cord blood did not differ between the groups, with the exception of tPAag concentrations, which tended to be numerically lower in the intervention group compared with the control group (Table 4; a two-sample *t*test on lognormal-transformed values of tPAag gave a *P*value of 0.05 for the difference between the groups).

## Discussion

**Figure 4:**
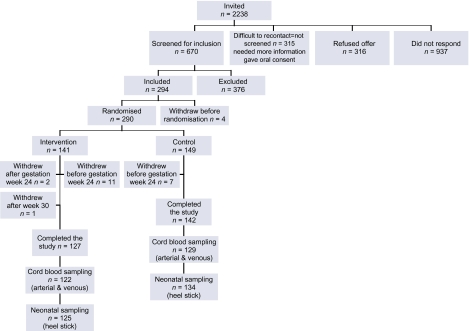
Trial profile.^9^

In this randomised controlled trial (Figure 4) of healthy white European subjects carrying singleton pregnancies, we found no effect of an antiatherogenic diet on maternal concentrations of sCAMs, CRP and haemostatic parameters (PAI-1, PAI-2, tPAag and vWF) between the dietary intervention and control groups. Moreover, no differences were found between the groups in concentrations of the fetal blood variables measured other than a tendency to lower concentrations of tPAag in the intervention group. Thus, the previously reported preventive effect of the current dietary intervention on preterm delivery is not likely to involve modification of systemic inflammatory biomarkers.

To our knowledge, the effect of a dietary intervention on markers of endothelial activation and inflammation has not been studied previously in women of fertile age or during pregnancy. The reported significant differences in dietary saturated and unsaturated fat intakes between the groups are supported by the observed difference in some serum fatty acid levels (myristic, eicosapentaenic) between the intervention and control groups. Dietary counselling, similar to the advice given to the intervention group in our study but which extended over a 3-year interval, has been shown to reduce ICAM and tPAag concentrations in a randomised trial of men aged 65–75 years.^29^

The lack of effect of an antiatherogenic diet in pregnancy on markers of endothelial activation and inflammation may be limited to the current study population, which consisted of healthy, nonsmoking pregnant women with no previous pregnancy complications. There is also a possibility of type II error since power analysis was carried out in regard to the primary hypothesis i.e. reducing maternal and neonatal total cholesterol levels.^9^ The major strength of the CARRDIP study, on the other hand, is that it is a randomised dietary intervention trial in pregnancy with a prospective design. In addition, the different dietary intake between the groups was verified through characterisation of the serum fatty acid profile.

Maternal concentrations of low-density lipoprotein (LDL) cholesterol increased through gestation as expected,^9^ but the observed decrease in LDL cholesterol concentrations in the intervention arm^9^ was not accompanied by lower CRP concentrations in this group. It is, therefore, notable that the significant increase in all other systemic parameters of inflammation through the study period, in accordance with previous literature,^12^,^15^,^20^,^22^ was not observed with regard to CRP concentrations, which remained essentially unchanged (Figure 2d). We have not identified any previously published longitudinal studies of CRP levels in pregnancy. CRP concentrations at 13 weeks of gestation are highly correlated with pre-pregnancy adiposity.^31^ Our findings concerning the significant positive correlation between baseline CRP and baseline BMI in the entire cohort suggest that adiposity is a factor influencing the level of CRP in pregnancy, as seen in nonpregnant populations.

In normal pregnancy, concentrations of most clotting factors are reported to increase,^32^ and our results agree with previous observations. Fibrinolytic activity diminishes as tissue plasminogen activator activity decreases, probably due to the increase in the plasminogen activator inhibitors PAI-1 and PAI-2.^32^ We observed increases in concentrations of tPAag, vWF, PAI-1 and PAI-2 from baseline to week 36 in both the control and intervention groups. However, the increase did not differ between the groups. This finding indicates that lowering total and LDL cholesterol concentrations in low-risk pregnancies by lowering dietary saturated fat^9^ is not accompanied by changes in haemostatic variables, at least not within the range of LDL lowering achieved in the present study.

Inflammatory processes have been shown to take place locally in the uteroplacental unit, especially in pre-eclampsia.^33^ Such local inflammatory processes may also occur in some cases of preterm delivery, since it has been shown that failure of physiological transformation and atherosis of the uteroplacental spiral arteries are often seen in cases of preterm delivery and bear several similarities to atherosclerosis in general, including lipid deposition and inflammatory features.^3^,^4^ Moreover, increased concentrations of ICAM-1 in cervicovaginal fluid have been considered as a potential predictor of preterm delivery in women with symptoms of preterm labour.^34^ Although we did not find significant changes in the blood concentrations of the measured inflammatory markers, we cannot exclude the possibility that the dietary intervention somehow may have lead to changes in the local uteroplacental or cervical inflammatory response involving, for example, less enhanced leukocyte/endothelial interaction. Whether lowering of maternal LDL cholesterol is directly involved in initiating a local anti-inflammatory response remains a hypothesis.

Another finding, which supports the notion of a possible local effect in the intervention group, is that we observed a tendency towards lower concentrations of fetal tPAag in cord blood of fetuses born to mothers in the intervention compared with the control group. To our knowledge, cord blood tPAag concentrations are not described in the literature. tPAag is regarded a marker of atherosclerotic activity and to be an independent marker of coronary heart disease.^25^,^35^ The effect of the diet on concentrations of tPAag in fetal blood is in accordance with the intervention study of Hjerkinn *et al.*^29^ However, we suggest that future studies attempting to assess a possible local effect of the intervention diet should include power calculations for tPAag in cord blood as a primary hypothesis.

In conclusion, we found no effect of an antiatherogenic diet during pregnancy on biomarkers of endothelial activation, inflammation and haemostasis. All biomarkers increased during the study period except for CRP, which was stable. Thus, the previously reported effects of the diet during pregnancy on maternal lipid profile and risk of preterm delivery do not seem to involve changes in the systemic inflammatory responses of pregnancy.
